# Supplementary material for: Ultrahigh drive current and large selectivity in GeS selector
Source: Nat Commun. 2020 Sep 15;11:4636. doi: 10.1038/s41467-020-18382-z (PMC7493911; doi:10.1038/s41467-020-18382-z)
Supplement: Supplementary file 1 — Supplementary Information [file 41467_2020_18382_MOESM1_ESM.pdf]

Supplementary Information

Ultrahigh Driving Current and Large Selectivity in GeS Selector

Jia et al.
*e-mail: minzhu@mail.sim.ac.cn

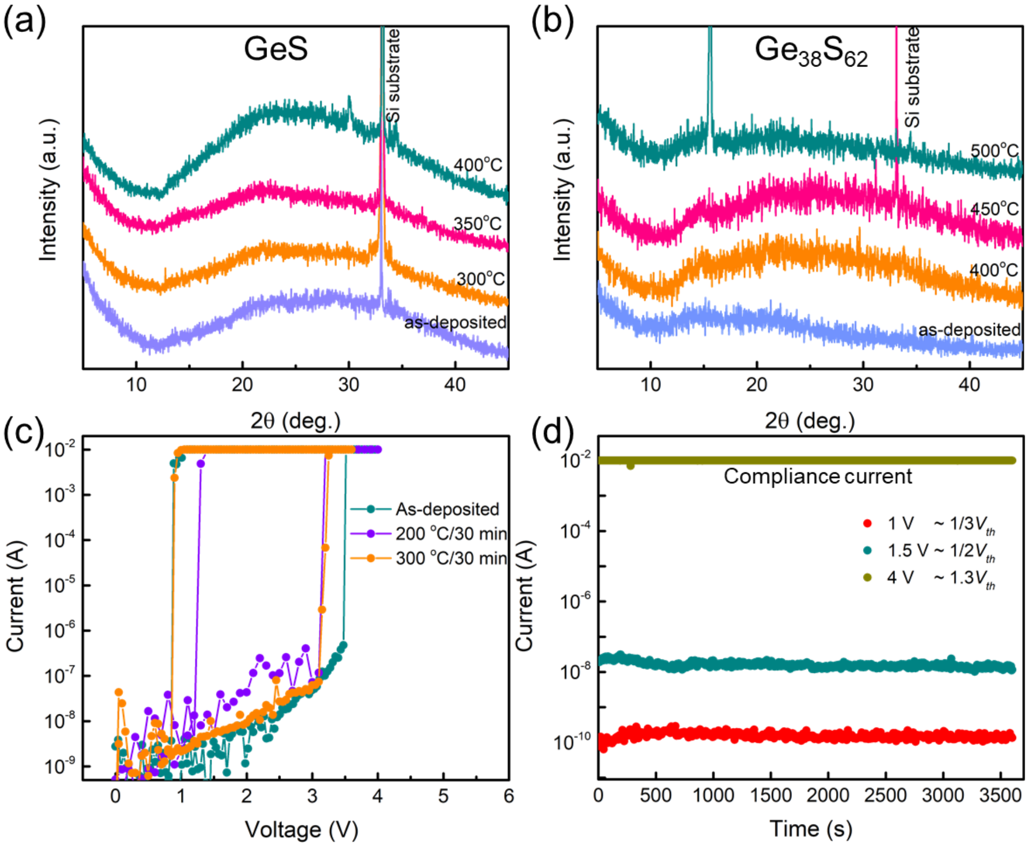

Supplementary Figure 1 | Thermal stability of GeS film and device. XRD patterns of annealed a, GeS and b, $Ge_{38}S_{62}$ films at different temperatures. c, *I-V* curves of the GeS-based cell annealed at 200 °C and 300 °C for 30 min. d, DC stress test of GeS-based OTS cell by applying 1 V (1/3 V_{th}), 1.5 V (1/2 V_{th}) and 4 V (1.3 V_{th}) voltages. 200 nm-thick GeS and $Ge_{38}S_{62}$ were deposited on SiO_2/Si substrate annealed at different temperatures for 30 min. A small amount of GeS crystalline phase appears in the film annealed at 400 °C, while the sample at 350 °C is completely amorphous. Hence, the crystallization temperature is higher than 350 °C, which is higher than reported mature selection material systems-GeSe[1]. A crystallization temperature higher than 450 °C is observed for $Ge_{38}S_{62}$.

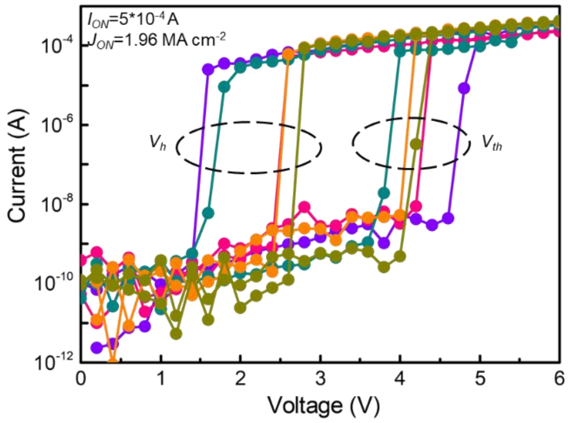

Supplementary Figure 2 | The *I-V* characteristics of GeSe OTS device with the same device

structure. Low drive current, large hold voltage, poor selectivity and variability of GeSe device can be obtained by the *I-V* sweep.

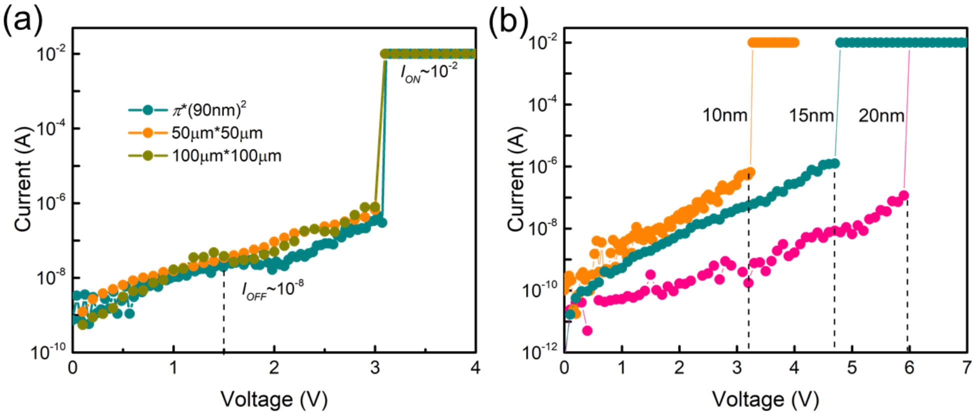

Supplementary Figure 3 | The *I-V* characteristics of different GeS devices. a, device size. b, GeS thickness. The electrical parameters are independent on the size of device and sensitive to the thickness. The threshold voltage increases proportionally to the thickness, leading to the same threshold electric field.

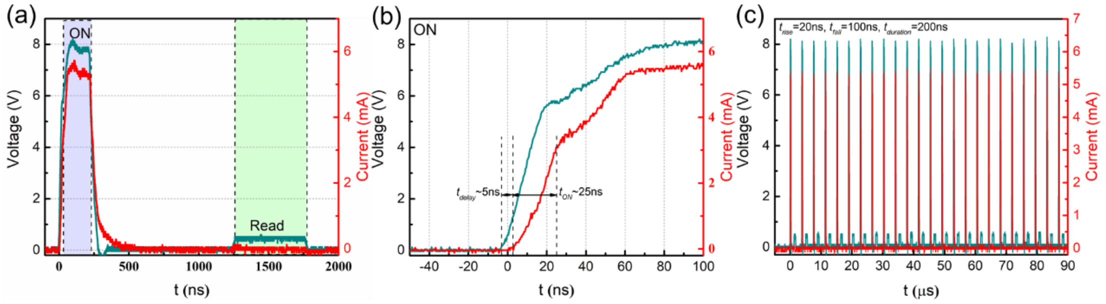

Supplementary Figure 4 | The transient response of GeS device under the square pulse. a, Dynamical response of device under 8 V/200 ns programming pulse with a rising edge of 20 ns and 0.5 V /500 ns read square pulses. The interval of between them is 2 μs. b, Close-up view of turn on speed. c, Dynamical characteristics under multiple nanosecond pulses applied to a GeS device. The device turns on at high pulse within 30 ns including the delay time of 5 ns, the current of which enable to reach ~mA. A subsequent small pulse is used to examine whether the device was successfully turn off after the voltage was removed. Under the stimulus of multiple nanosecond pulses the device can successfully realize the volatile functionality.

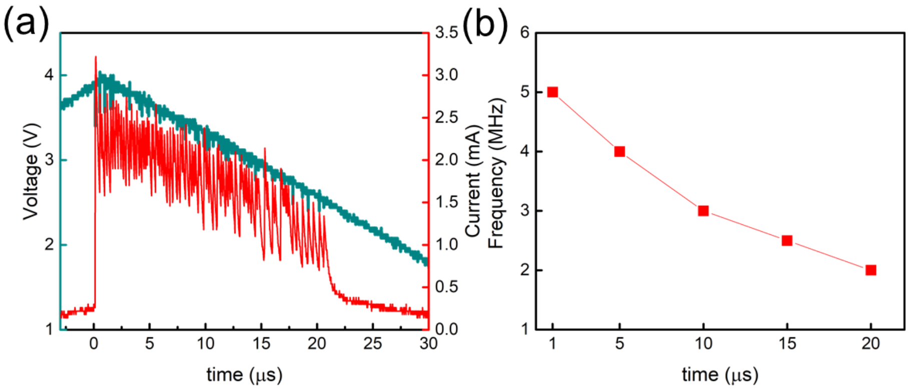

Supplementary Figure 5 | Dynamical response characteristics of neuronal circuit. a, The circuit response under a triangle pulse voltage. b, Corresponding frequency versus time curve. The data shows the frequency of the output response as the voltage successively decreases under the triangular pulse with near-V_{th} amplitude in neuronal circuit. The response frequency is decreasing with the voltage decrease.

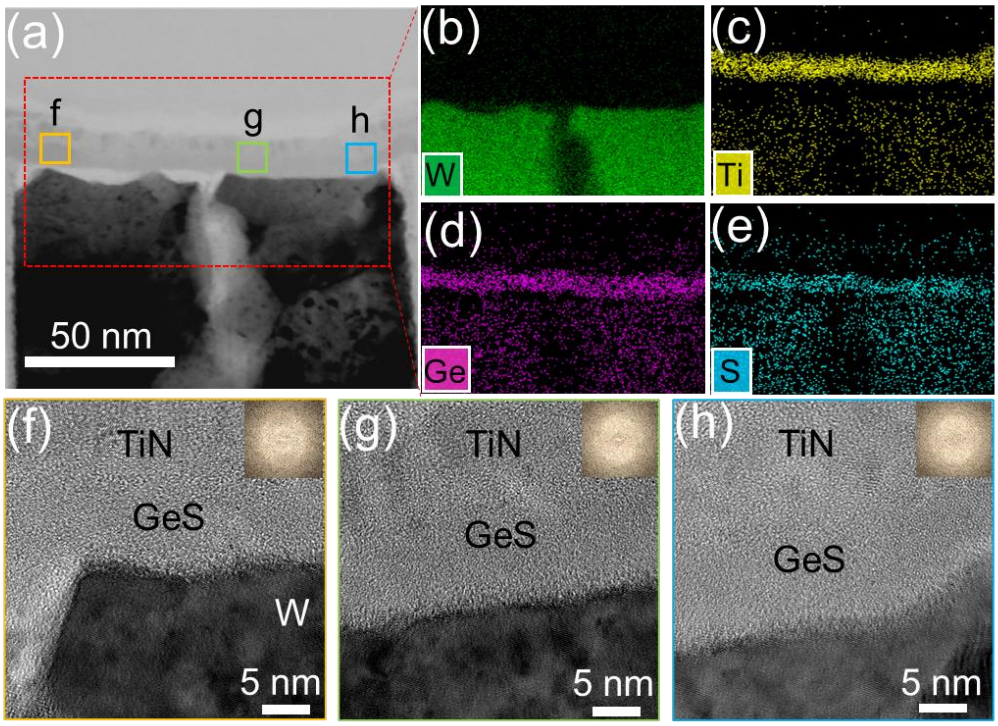

Supplementary Figure 6 | The microstructure of device undergoing electrical operations. a, The HAADF image of GeS-based device. b-e, Corresponding EDS element mappings of W, Ti, Ge and S respectively. f-h, Cross-section HRTEM images. Insets are Fast Fourier transform image of GeS layer. This GeS device has undergone repeated electrical operations. Homogenous element distributions of GeS film, without metal filament, are observed in the repeatedly operated selectors, as shown in **Supplementary Figure 6** a-e. Also, the GeS film maintains its amorphous state (**Supplementary Figure 6** f-h), which proves that the threshold switching of these OTS selectors is the result of electronic processes, unlike the Conductive bridge threshold switch (CBTS)[2] and Phase change memory (PCM)[3].

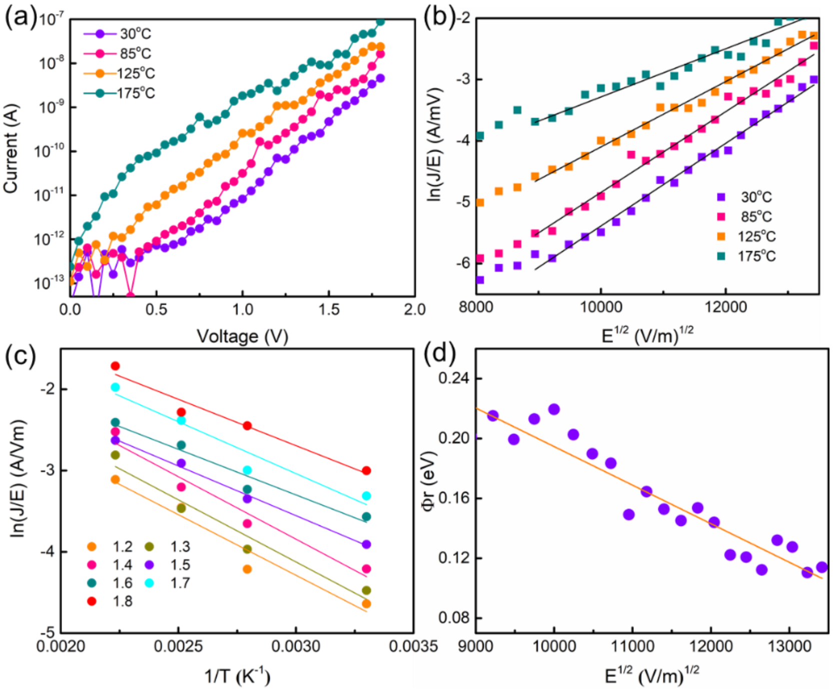

Supplementary Figure 7 | Poole-Frenkel fitting model showing the trap depth is 0.45eV. a, *I-V* performances of GeS selector at various temperatures. b, *In(J/***E***)* vs. $\mathbf{E}^{1/2}$. c. *In(J/E)* vs. *1/T*. d, Φ_r vs. $\mathbf{E}^{1/2}$. Both *In (J/***E***)* vs. $\mathbf{E}^{1/2}$ and *ln (J/***E***)* vs. *1/T* exhibit good linear relationships, fitting well with Poole-Frenkel model[4]. Linear dependence of the trap depth on the applied electric field, from which a trap depth of ~0.45 eV is extracted, which is particularly consistent with the experimental PDS results.

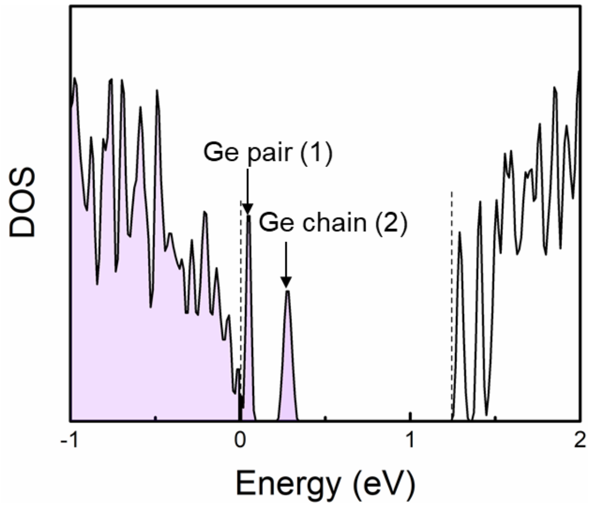

Supplementary Figure 8 | DOS of amorphous GeS without Ge vacancy.

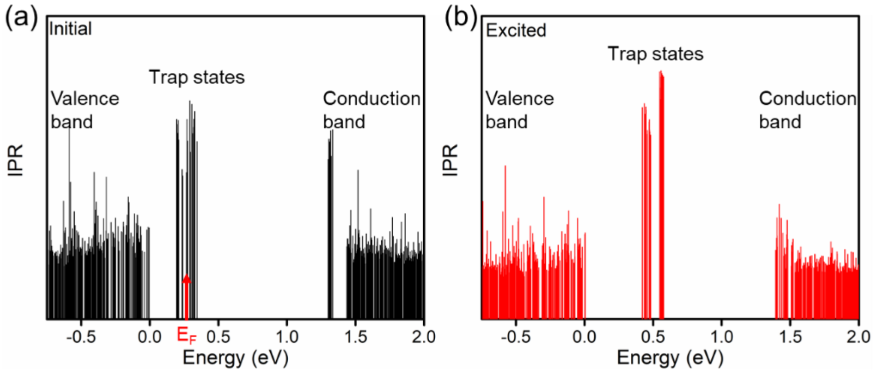

Supplementary Figure 9 | Inverse participation ratio (IPR) for amorphous GeS before and after hole excitation.

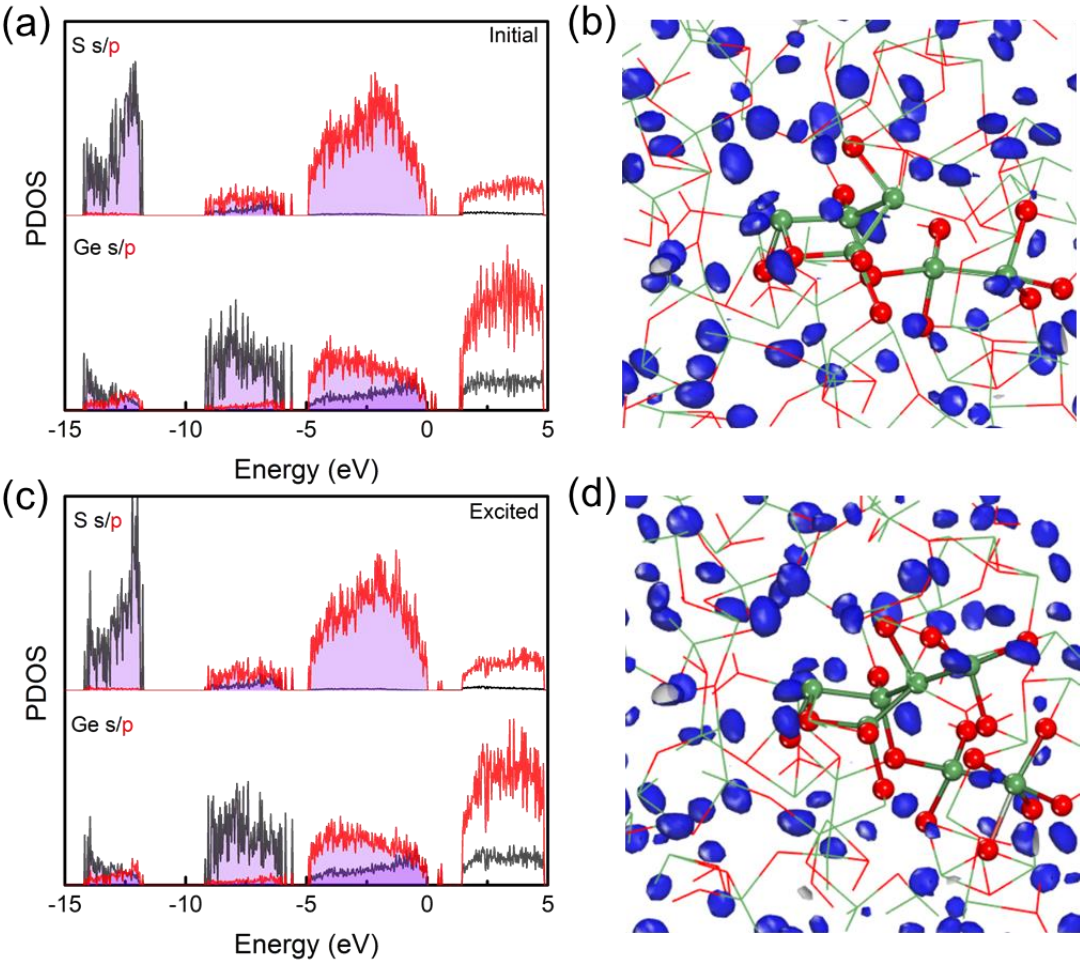

Supplementary Figure 10 | a, c Projected density of states (PDOS) on S and Ge and b, d Isosurface of electron localization function of amorphous GeS in the ground state and excited state, respectively.

Supplementary Reference

1. Govoreanu, B. *et al.* Thermally stable integrated Se-based OTS selectors with >20

MA/cm^2 current drive, >3.10^3 half-bias nonlinearity, tunable threshold voltage and excellent endurance. in *2017 Symp. on VLSI Technology (VLSIT)* **21**, T92–T93 (IEEE, 2017).
2. Midya, R. *et al.* Anatomy of Ag/Hafnia-based selectors with 10^{10} nonlinearity. *Adv. Mater.* **29**, 1604457 (2017).
3. Xia, M. *et al.* Ti-Sb-Te alloy: A candidate for fast and long-life phase-change memory. *ACS Appl. Mater. Interfaces* **7**, 7627–7634 (2015).
4. Ielmini, D. & Zhang, Y. Analytical model for subthreshold conduction and threshold switching in chalcogenide-based memory devices. *J. Appl. Phys.* **102**, 054517 (2007).
